# Prevalence and Infection Intensity of Soil‐Transmitted Helminths and Schistosomiasis Among Preschool Children in Mainland Tanzania

**DOI:** 10.1155/japr/6492754

**Published:** 2026-04-22

**Authors:** Kaunara Azizi, Analice Kamala, Devotha Mushumbusi, Francis Modaha, Tuzie Edwin, Ramadhani Mwiru, Mwanaidi Kafuye, Peter Kaswahili, Donath Tarimo, Germana Leyna

**Affiliations:** ^1^ Department of Food Sciences and Nutrition, Tanzania Food and Nutrition Centre, Dar es Salaam, Tanzania; ^2^ Child Nutrition and Development Section, UNICEF, Dar es Salaam, Tanzania, unicef.org; ^3^ Department of Laboratory Sciences, National Institute for Medical Research, Dar es Salaam, Tanzania, nimr.mrc.ac.uk; ^4^ Nutrition Unit, Ministry of Health, Dodoma, Tanzania, moh.koshi.gov.np; ^5^ Department of Parasitology and Medical Entomology, Muhimbili University of Health and Allied Sciences, Dar es Salaam, Tanzania, muchs.ac.tz; ^6^ Department of Community Health and Nutrition, Tanzania Food and Nutrition Centre, Dar es Salaam, Tanzania; ^7^ Department of Epidemiology and Biostatistics, Muhimbili University of Health and Allied Sciences, Dar es Salaam, Tanzania, muchs.ac.tz

**Keywords:** preschool children, schistosomiasis, soil-transmitted helminths, Tanzania, worm infestation

## Abstract

Soil‐transmitted helminths (STHs) and schistosomiasis (SCH) are significant public health concerns in Tanzania, known to impair cognitive and physical development in young children. To effectively guide control programs, a comprehensive understanding of their current prevalence and intensity is essential. This cross‐sectional study was aimed at determining the prevalence and intensity of STH and SCH infections and associated anemia among preschool children aged 12–59 months across seven agroecological zones in Tanzania. A total of 1612 children were examined using the Kato‐Katz technique for *Schistosoma mansoni* and STH eggs, urine filtration for *Schistosoma haematobium* eggs, and the HemoCue photometer for anemia. Statistical associations were assessed using chi‐square tests (*p* < 0.05). The prevalence of any STH infection was 4.82% (95% CI: 3.84–6.02), with hookworm being the most prevalent (3.56%; 95% CI: 2.63–4.55), followed by *Ascaris lumbricoides* (1.19%; 95% CI: 0.65–1.79) and *Trichuris trichiura* (0.07%; 95% CI: 0.01–0.37). The overall SCH prevalence was 0.90%, and 34.06% of children were anemic (95% CI: 31.78–36.41). All STH infections were of mild intensity, and among SCH cases, infection intensity was predominantly light, with only one child exhibiting a heavy *S. mansoni* infection. Children residing in rural areas exhibited a 2.57‐fold higher likelihood of helminth infection compared to urban children (*p* < 0.001). While 85.2% of parents reported knowledge of helminthiasis, only 57.57% reported a history of deworming. The observed low prevalence of STH and SCH suggests a potential positive impact of the national deworming program. However, ongoing surveillance and regular evaluations are crucial to monitor program effectiveness, address persistent anemia, and optimize intervention strategies.

## 1. Introduction

Helminth infections caused by soil‐transmitted helminths (STHs) and schistosomiasis are prevalent infestations in the developing world [1]. The associated morbidity is primarily linked to heavy‐intensity infections. Approximately 1.45 billion people worldwide are estimated to be infected with STH parasites, resulting in 4.98 million disability‐adjusted life years lost and 5.18 million years lost due to disability [2]. Children are most vulnerable to these infections, experiencing a range of pathological consequences such as gastrointestinal symptoms, malnutrition, general weakness, and impaired growth and development [3, 4].

STH detrimentally impacts the nutritional status of infected individuals through various mechanisms, including blood feeding, leading to iron and protein loss, increased malabsorption of nutrients, potential competition for vitamin A in the intestine, and decreased appetite, resulting in reduced nutritional intake and physical fitness [5, 6]. Due to the overlap of these infections and their effects on children′s health, the World Health Organization (WHO), World Bank, and other United Nations agencies, bilateral, and civil society organizations have collaborated to integrate STH and schistosome control through targeted, school‐based deworming programs [7, 8].

Hookworm infections above 20%–30% are considered endemic, and when associated with anemia prevalence, hookworm control should be incorporated into strategies aimed at improving the health and nutritional status of children, girls, and women, as evidenced by existing literature [9]. Despite national control efforts, areas of ongoing transmission still exist, especially in communities with limited access to sanitation and preventive treatment. Furthermore, research suggests a high prevalence of schistosomiasis in Tanzania, with infection rates exceeding 90% among school‐aged children, particularly in regions bordering Lake Victoria [10, 11]. This persistent burden is compounded by ecological overlap, where transmission hotspots for both STH and SCH converge, amplifying the risk of coinfection and complicating intervention strategies [12].

In 2001, the WHO passed a resolution (WHA54.19) for member states to deworm all children under 5 years of age once or twice a year, depending on the prevalence of intestinal worm infection (20% or above and 50% or above, respectively), to minimize its consequences [13, 14]. In line with this resolution, the government of Tanzania has integrated mass deworming with vitamin A supplementation for children aged 12–59 months since 2004, with coverage above 90%. Despite the recognized burden of these parasitic infections, limited data exist on the current prevalence and intensity of STH and SCH among preschool children in Tanzania, hindering effective program planning and evaluation.

This study is aimed at determining the prevalence and intensity of STH among children aged 12–59 months in mainland Tanzania. As STH is coendemic with SCH in Tanzania, it was deemed necessary to include schistosomiasis in the survey. This information is important for strengthening the understanding of national STH and SCH transmission patterns, which in turn will be used in developing effective, targeted, and evidence‐based prevention and control interventions.

## 2. Methodology

### 2.1. Study Design, Population, and Inclusion and Exclusion Criteria

This was a community‐based cross‐sectional study. The study population was preschool children aged 12–59 months and their parents/caretakers. Inclusion criteria were all children aged between 12 and 59 months and whose parent/caretaker consented to participate. Those who had an infectious illness, either by self‐reporting or medical records, were unable to provide stool and urine samples at the time of data collection, or whose parent/caregiver was unable to provide their information, were excluded from the study.

### 2.2. Study Area, Sampling Procedure, and Sample Size

The survey was conducted in seven agroecological zones of mainland Tanzania in December 2022. A multistage cluster sampling technique was used. First, one region was selected from each of the seven agroecological zones using probability proportional to size. Within each region (Coast, Dodoma, Kigoma, Kilimanjaro, Lindi, Mbeya, and Mwanza), councils were stratified into urban and rural, and one council from each stratum was randomly selected. From each selected council, one ward was randomly chosen, followed by the random selection of two enumeration areas (streets or villages) per ward. Fifteen households with at least two children aged 12–59 months were randomly selected from each enumeration area. This process yielded a target sample of 1680 children from 840 households. The sample size was determined using the following formula:

n=Z2P1−Pd2

where *n* is the sample size, *d* is the margin of error, *Z* is the statistical value for a level of 95% confidence interval (CI) (1.96), and *P* is the proportion of preschool children with STH. With the STH prevalence (*P*) of 9% [15] among preschool children, confidence level of 95%, margin of error of 1.5%, and estimated population of 8,000,000–10,000,000 preschool children, the calculated sample size was 1398. Taking into consideration a 20% nonresponse rate, the total sample size was 1678 and rounded up to 1680.

### 2.3. Recruitment of Study Participants

A total of 840 households with at least two eligible children (12–59 months) in each household were randomly selected from a list of all households with eligible children in every selected enumeration area (hamlet/street). A list of all households with eligible children was obtained from the office of the village executive officer (VEO). The age of the child was verified using relevant documents, including a birth certificate, health card/clinic card, child health booklet, vaccination card, or booklets with the name of the child and date of birth. The study teams kept records of the number of eligible households (with at least two children aged 12–59 months) in each selected enumeration area, the number of parents/caretakers consenting, the number of parents/caretakers declining for the study, and reasons for declining.

### 2.4. Data Collection

The study involved the quantitative data collection, which involved administering a semistructured questionnaire to all consented parents/caretakers of eligible children in all enumeration areas and collecting specimens (blood, stool, and urine) from all participating children. Data collected using a questionnaire included social–demographic characteristics, knowledge regarding STH, risk factors for worm infestation, and history of previous deworming treatment received.

### 2.5. Stool, Urine, and Blood Sample Collection, Processing, and Examination

#### 2.5.1. Specimen Collection

Each eligible child for the study was assigned a specific identification and provided with a labeled stool container with tight covers bearing serial numbers of the subjects and was requested to collect about 5 g of stool the following morning. All the stool samples were received at an organized central place and processed on the same day using a direct saline wet mount for motile intestinal parasites. The study participants were given a labeled plastic container (100 mL capacity) and asked to provide a urine sample. About 10 mL of the participant′s urine sample was processed by the urine filtration method and microscopically examined for *Schistosoma haematobium* eggs. Urine samples were successfully collected from 1564 children for schistosome examination, and 1516 stool samples were available for STH examination. A blood sample from a finger prick was taken for evaluation of hemoglobin levels. The finger was cleaned with alcohol swabs before pricking. The first drop of blood was wiped off with cotton wool, while the second drop was placed into a microcuvette using safety lancets and screened for anemia using the HemoCue (HemoCue Hb 301+) photometer. Blood samples were available for all 1612 study participants.

#### 2.5.2. Examination of STH and *Schistosoma mansoni* Using the Kato‐Katz Technique

Stool samples were processed using the Kato‐Katz smear technique, with all thick‐smear slides prepared and read on the same day as collection under the light microscope at X10 and X40 magnifications. The slides were read by two laboratory technicians within 1 h of preparation to identify hookworm eggs and then read again for *Ascaris lumbricoides* and *Trichuris trichiura* eggs 1–2 h after slide preparation to allow the slides to clear. Examination for *S. mansoni* eggs was done independently a few minutes later. The egg counts on each slide were done to get the number of egg counts per slide. In the event of discrepancies between the readings of two technicians, a third technician was consulted in order to reach a consensus. For quality assurance, a random sample of 10% of the negative and positive Kato‐Katz thick smears was reexamined by a third laboratory technician. A small portion of the stool samples was also preserved in 10% formalin for repeating the tests whenever required. The infection intensity of STH and *S. mansoni* was estimated by multiplying the total number of eggs counted by 24, which gives the eggs per gram (EPG) of stool. The infection intensity was classified according to WHO guidelines [16].

#### 2.5.3. Examination of *S. haematobium* Infection

Collected urine samples were grossly examined for macrohematuria. Urine dipstick/urinalysis reagent strips (MISSION, Expert, United States) were used to determine microhematuria. A filtration technique was used to screen urine samples, and a light microscope was used to examine urine filters for the presence of *S. haematobium* eggs (WHO, 1991). Each sample was examined independently by two clinical laboratory technicians, and at the end of each fieldwork day, 10% of all the positive and negative samples were reexamined. The infection intensity was classified according to WHO guidelines [16].

#### 2.5.4. Screening for Anemia

Fingers were cleaned with alcohol swabs before pricking. The first drop of blood, soon after pricking, was wiped off with cotton wool while collecting the second drop into a microcuvette using safety lancets, and the blood was screened for anemia using the HemoCue (HemoCue Hb 301+) photometer. The microcuvette with the blood sample was loaded into the calibrated HemoCue photometer, and the Hb concentration was read to the nearest 0.1 g/dL. All children with an Hb level <11 g/dL were considered anemic. Children with Hb from 10 to 10.9 g/dL were graded as mild, those with Hb between 7 and 9.9 g/dL were graded as moderate, and those with Hb < 7 g/dL were graded as severe anemia [17].

#### 2.5.5. Hb Adjustments

Corresponding altitudes of all enumeration areas were determined so that the observed individual child′s hemoglobin level and cut‐off for anemia were adjusted according to WHO guidelines [18].

### 2.6. Data Management and Statistical Analysis

An individual semistructured questionnaire was assigned a unique identifier number (bar code number) that was linked to laboratory data for the same individual child. The data collected through a semistructured questionnaire was checked for completeness and linked to the corresponding laboratory examination results. Sampling weights were applied, and prevalence estimates were calculated. Free‐text data were translated into English and coded into categories before being analyzed.

Data were analyzed using Stata Version 18 (STATA Corporation, College Station, TX, United States). Descriptive statistics were calculated as proportions with 95% CIs. Bivariate associations between helminth infection and independent variables (sex, age, residence, and relation with caretaker) were assessed using chi‐square tests. To account for potential confounding, multivariable logistic regression was employed to identify factors independently associated with STH infection. Results were considered significant at *p* < 0.05. Infection intensity results were grouped according to the WHO‐recommended intensity groupings into the light, moderate, and heavy infection intensities [16].

## 3. Results

### 3.1. Social–Demographic of the Study Participants

The distribution of social–demographic characteristics of the children and parents/caretakers is summarized in Table 1. Of the 1680 children and 840 parents/caretakers invited to participate, complete and valid data were obtained from 1612 children and 806 parents/caretakers, which formed the final analytic sample. The primary reasons for exclusion were the inability to provide stool or urine samples and withdrawal of consent. The age of the survey children ranged from 12 to 59 months; 50.1% of the children were girls and 49.9% were boys. About 61% of parents/caretakers were male, and most were in the range of 18–49 years of age. More than three‐quarters (83.4%) of parents/caretakers were married, and more than half (61.7%) had a primary education level.

**Table 1:** Social–demographic characteristics of children, parents/caretakers, and household characteristics.

Variables	Number (*n*)	(%)
Children aged 12–59 months (*N* = 1612)		
Gender		
Male	804	49.9
Female	808	50.1
Age		
12–23	416	25.8
24–59	1196	74.2
Region		
Dodoma	240	14.9
Kigoma	230	14.3
Kilimanjaro	238	14.8
Lindi	236	14.6
Mbeya	208	12.9
Mwanza	224	13.9
Pwani	236	14.6
Residence type		
Urban	818	50.7
Rural	794	49.3
Relation with caretaker		
Parents	1075	66.7
Other	537	33.3
Parent/caretaker characteristics (*n* = 806)		
Age^b^		
18–29	218	27.7
30–39	273	34.6
40–49	163	20.7
≥ 50	134	17.0
Gender		
Female	314	39.0
Male	492	61.0
Marital status		
Single/ever married^a^	134	16.6
Married/cohabit	672	83.4
Education level		
None	156	19.0
Primary education	497	62.0
Secondary education	114	14.0
Tertiary education	21	3.0
University education	18	2.0
Household income activity^b^		
Farmer/livestock	466	60.6
Entrepreneur/informal	260	33.8
Formal employment	43	5.6

^a^Ever married included those who are widowed/divorced.

The total value in all variable are equal (1516)

### 3.2. Prevalence of STH and SCH Infections

Of the total surveyed children, 4.82% (95% CI = 3.8–6.0) (Table 2) and 0.9% (95% CI = 0.49–1.49) (Table 3) had at least one type of STH infection and SCH, respectively, with mild intensity. Of all detected STH, hookworms were the most prevalent (3.56%). For schistosomes, *S. mansoni* was the most prevalent (0.64%). Children residing in rural areas had more than double the odds of STH infection compared with those in urban areas (adjusted OR: 2.45; 95% CI: 1.45–4.14). Regional variation was also pronounced, with children from Lindi showing the highest odds of infection relative to Dodoma (adjusted OR: 10.92; 95% CI: 3.69–32.32), while no infections were recorded in Kilimanjaro. In contrast, sex, age group, and caretaker relationship were not significantly associated with STH infection in either the bivariate or multivariate analysis (Table 4). Only a small fraction (1.25%) of the 806 surveyed households had both children present with STH, while an even smaller fraction (0.25%) had both children present with schistosome.

**Table 2:** Types of STH and prevalence and severity of infection among children 12–59 months (*N* = 1516).

Types of STH infection	Severity of infection			
	Severe *n* (%)	Moderate *n* (%)	Mild *n* (%)	95% CI
Ascariasis	0	0	18 (1.19)	0.65–1.79
Trichuriasis	0	0	1 (0.07)	0.00–0.37
Hookworms	0	0	54 (3.56)	2.63–4.55
All STHs	0	0	73 (4.82)	3.8–6.0

**Table 3:** Prevalence and severity of schistosome infection among children 12–59 months (*N* = 1564).

Types of schistosome	Severity of infection				
	Light	Moderate	Heavy	Total *n* (%)	95% CI
*S. haematobium*	4	n/a	0	4 (0.26)	0.30–1.17
*S. mansoni*	7	2	1	10 (0.64)	0.07–0.65
All SCHs				14 (0.90)	0.49–1.49

**Table 4:** Factors associated with STH infection among children 12–59 months (*N* = 1516).

Variable	Examined (*N*)	Positive for STH *n* (%)	Unadjusted OR (95% CI)	Adjusted OR (95% CI)^a^	*p* value
Sex					
Male	762	37 (4.9)	1.00 (ref)	1.00 (ref)	0.941
Female	754	36 (4.8)	0.98 (0.61–1.57)	0.97 (0.60–1.56)	
Age of the child (years)					
< 2	399	17 (4.3)	1.00 (ref)	1.00 (ref)	0.547
2–5	1117	56 (5.0)	1.18 (0.68–2.06)	1.15 (0.66–2.02)	
Relation with caretaker					
Parents	1007	49 (4.9)	1.00 (ref)	1.00 (ref)	0.897
Other	509	24 (4.7)	0.97 (0.59–1.60)	0.98 (0.59–1.62)	
Settings					
Urban	757	21 (2.8)	1.00 (ref)	1.00 (ref)	< 0.001
Rural	759	52 (6.9)	2.60 (1.55–4.36)	2.45 (1.45–4.14)	
Region					
Lindi	235	39 (16.6)	11.84 (4.04–34.70)	10.92 (3.69–32.32)	< 0.001
Kilimanjaro	229	0 (0)	N/A	N/A	
Mbeya	198	9 (4.5)	2.85 (0.86–9.44)	2.67 (0.80–8.90)	
Kigoma	215	8 (3.7)	2.36 (0.69–8.04)	2.20 (0.64–7.54)	
Pwani	217	13 (6.0)	3.93 (1.21–12.79)	3.71 (1.13–12.14)	
Mwanza	194	3 (1.5)	0.94 (0.21–4.16)	0.88 (0.20–3.94)	
Dodoma	228	1 (0.4)	1.00 (ref)	1.00 (ref)	

*Note:* The Kilimanjaro Region had zero cases; thus, odds ratios could not be estimated in the logistic model and were excluded from the multivariate analysis.

Abbreviations: CI, confidence interval; OR, odds ratio; STHs, soil‐transmitted helminths.

^a^Adjusted for all variables in the table (sex, age, caretaker, setting, and region) to control for potential confounding.

^∗^Totals differ due to missing values in some variables.

### 3.3. Infection Intensity/Severity Levels

Infection intensity for STH was uniformly mild across all positive cases. The geometric mean egg counts were 48 EPG for hookworm (range: 24–120 EPG), 72 EPG for *A. lumbricoides* (range: 24–144 EPG), and 24 EPG for *T. trichiura*. For *S. mansoni*, mean intensity among positive cases was 96 EPG (range: 24–480 EPG), with only one child classified as having a heavy infection (480 EPG). All *S. haematobium* infections were light (≤ 50 eggs/10 mL urine).

### 3.4. Water, Sanitation, and Hygiene (WASH) Practices

Results from the survey indicated that the main source of drinking water was from the public tap (23.9%), as detailed in Table S1. Water from wells was another frequent source of drinking water, while spring and rainwater were rare sources. Regarding sanitation facilities, 83.4% of surveyed households do not share toilets. When the sources of drinking water and sanitation facilities were categorized according to WHO/UNICEF guidelines (i.e., improved or unimproved), overall 87.3% of respondents reported obtaining water from an improved source, and 46.7% reported utilizing improved sanitation facilities. On the other hand, almost less than half (42.7%) of children wore shoes when visiting the toilet at all times, while 28.3% did not wear shoes at all. Of those responding, 46.6% of children washed their hands after visiting the toilet, and 59.0% washed their hands using detergent/soap. About 50% of children defecate in the toilets, 20.6% use special utensils (potty), 15.1% defecate in any place surrounding the house, and 10% use assisted potting (ground/floor) (Table S1). Due to low prevalence, it was not necessary to associate the risk factors (WASH) with infection rate or intensity.

### 3.5. Knowledge on STH, History of Vitamin A Supplementation, and STH and SCH Treatment

About 85.2% and 57.3% of respondents had knowledge about STH and schistosomiasis, respectively. The majority of the respondents (80.6%) got information on STH from a healthcare worker. The history of deworming treatment was reported by 57.57% of respondents, and 14.58% did not remember. Similarly, 54.96% and 15.41% of respondents reported their children to have received deworming tablets within 0–3 and ˃ 6 months, respectively, before the survey. About 81.64% reported their children to have received vitamin A supplementation in the previous 6 months (Tables S2 and S3).

### 3.6. Anemia Status Among Children Aged 12–59 Months

The results indicate that 34.1% of children who participated in the study were anemic, with varying degrees of severity. Only nine children (0.6%) had severe anemia (Table 5).

**Table 5:** Prevalence and level of anemia among children 12–59 months (*N* = 1612).

WHO cut‐off point (g/dL) for anemia	*n* (%)	95% CI
Severe anemia (< 7 g/dL)	9 (0.6)	0.26–1.06
Moderate anemia (7.0–9.9 g/dL)	184 (11.4)	9.90–13.06
Mild anemia (10.0–10.9 g/dL)	357 (22.1)	20.14–24.25
Total anemic	550 (34.1)	31.8–36.49

*Note:* Source: [17].

## 4. Discussion

This is the first survey conducted in seven agroecological zones of mainland Tanzania addressing the prevalence and intensity of STH and SCH infections among preschool children aged 12–59 months, which benefited from leveraging the approach of integrating a mass deworming program with vitamin A supplementation. The viability of integrating multiple programs in most developing countries is to optimize limited resources. The objective of this study was to fill the current gap in data on the prevalence and infection intensity of STH and schistosomiasis among preschool children in mainland Tanzania.

While our findings indicate that the overall prevalence and intensity of STH and SCH infections were low for both rural and urban settings, this finding must be interpreted within the broader epidemiological context of Tanzania. The country is known for coendemicity of STH and SCH, with specific ecological zones reporting STH prevalence as high as 100% and SCH prevalence ranging from 12.7% to 87.6% [9]. Moreover, studies conducted among Tanzanian children have documented a significant burden of helminth infections with a prevalence rate ranging from 10% to 80%, highlighting the widespread occurrence of worm infection in this population [19–21]. Most of the infections found in this study were mild, with low egg counts for hookworm, *Ascaris*, and *Trichuris*. While almost all cases of schistosoma were also light, the discovery of one child with a heavy *S. mansoni* infection serves as a vital reminder that serious cases still exist in the community. Even when infections do not reach heavy status, these light‐intensity burdens can quietly lead to chronic issues like anemia and poor nutrition, meaning they still require consistent attention and monitoring to protect long‐term health.

The comparatively low prevalence observed in our study may reflect the impact of sustainable public health interventions, including mass deworming administration implemented twice yearly by the government. This is because the survey was conducted in December 2022, 3 months after the mass deworming campaign (August 2022). In addition, more than 50% and 25% of respondents reported their children to have received deworming drugs 3 and 6 months, respectively, before the survey. The mass deworming probably contributed more than any other factor because, from the results, the low prevalence and intensity do not appear to be due to good hygiene practices or availability of sanitation facilities. The infested cases observed probably represented reinfested cases or those who had not used the deworming drugs. Regular deworming can play an important role in improving the health of individuals as well as the entire population. In children, deworming can boost the child′s immunity, help control infections, and increase nutritional uptake. Moreover, it also helps reduce worm infection that occurs in the community [22].

When we examined the factors associated with STH infection using multivariate logistic regression to adjust for potential confounders, the marked geographic and environmental disparities that emerged from the bivariate analysis remained strikingly evident. Children living in rural areas consistently faced a higher risk of infection than those in urban settings, a finding that likely reflects underlying differences in water quality, sanitation infrastructure, and housing conditions that are not captured by a simple urban–rural binary. This pattern aligns with the general understanding that rural environments often harbor a greater burden of worms due to a convergence of risk factors, including poor environmental hygiene, inadequate access to safe water, and suboptimal sanitation facilities [23].

Regional variation was equally pronounced; children from Lindi carried a substantially elevated risk compared with those from other regions surveyed, while the complete absence of infections in Kilimanjaro was particularly notable. Such geographical comparisons are essential for establishing an accurate epidemiological picture and, more importantly, for identifying priority areas where interventions can be most effectively targeted. In contrast, demographic factors such as sex, age, and whether the child lived with parents or other caretakers showed no meaningful association with infection after accounting for setting and region, suggesting that these characteristics are less important drivers of STH risk than broader environmental and contextual factors. WASH practices are generally associated with a reduced risk of STH infections of at least 33% [24]. However, the status of WASH observed in this study was much lower than WHO′s targets, which require 100% access to at least basic WASH in areas endemic for neglected tropical diseases in order to achieve targets 6.1 (safe drinking water) and 6.2 (sanitation and hygiene) of Sustainable Development Goal 6 [24]. Knowledge of these factors will guide and inform preventive activities and initiate government policies that will help in the control of STHs and SCH.

There is growing evidence that serum levels of multiple micronutrients, including vitamin A, are reduced by STH infection and some evidence that these effects can be reversed by deworming [25]. The integration of deworming with a vitamin A supplementation program has been successfully implemented in many countries [26, 27], including Tanzania. In this survey, over 80% of respondents reported their children to have received vitamin A supplementation in the previous 6 months. These findings emphasize the critical role of vitamin A supplementation in maintaining optimal health and highlight the importance of ensuring a sufficient intake for overall well‐being.

The present study assessed the prevalence of anemia among preschool children aged 12–59 months, revealing a substantial burden of 34.06% despite a relatively low prevalence of STH and SCH infections. While the association between helminth infections and anemia is well‐established [28], our findings suggest that factors beyond parasitic infections are significantly contributing to childhood anemia in Tanzania. In Tanzania, malaria remains a leading cause of childhood anemia, particularly in regions with perennial transmission. For instance, a study in Bagamoyo reported that up to 60% of anemia cases in young children were attributable to *Plasmodium falciparum* infection [29]. Additionally, dietary deficiencies in iron, folate, and vitamin B12 are common in resource‐limited settings and have been documented among Tanzanian children and pregnant women [30]. The low prevalence of STH and SCH, coupled with the high rate of anemia, strongly implicates dietary deficiencies as a primary driver. Iron, folate, and vitamin B12 deficiencies, known to be prevalent in resource‐limited settings [31], are likely major contributors. Limited access to iron‐rich foods, insufficient intake of fruits and vegetables (sources of folate), and potentially low consumption of animal products (sources of vitamin B12) could explain the observed anemia rates [32]. Other potential contributors include genetic hemoglobinopathies such as sickle cell disease and thalassemia, which are prevalent in parts of the country [33, 34], as well as chronic infections that may induce anemia of inflammation [35].

The observed anemia prevalence may be affected by confounding factors, which warrant thoughtful examination. Firstly, other parasitic infections, beyond STH and SCH, such as malaria, cannot be discounted. Malaria, endemic in many regions of Tanzania, is a well‐recognized cause of anemia in young children [29]. Secondly, genetic hemoglobinopathies, such as sickle cell disease and thalassemia, which are prevalent in some African populations [36], could contribute to anemia. These conditions may not be readily detectable through the methods employed in this study. Thirdly, chronic infections, including bacterial and viral infections, can lead to anemia of chronic disease [37].

The presence of underlying infections, particularly in resource‐limited settings with limited access to healthcare, may contribute to the observed anemia rates. Furthermore, socioeconomic factors, such as poverty, limited access to clean water and sanitation, and poor maternal nutritional status, can indirectly influence anemia prevalence by affecting dietary intake, infection rates, and overall health [38]. Future studies should consider incorporating comprehensive assessments of these potential confounding factors, including malaria screening, hemoglobin electrophoresis, and detailed dietary assessments, to provide a more nuanced understanding of the causes of anemia in this vulnerable population. Additionally, investigations exploring the interaction between these factors and the impact of the deworming program on anemia are crucial to inform targeted interventions and improve child health outcomes.

This study was strengthened by a representative sampling method across zones, which resulted in good statistical power and broad generalizability. This study, however, is subject to several limitations. The study utilized a cross‐sectional design, which is prone to selection bias and reverse causality; thus, causal relationships cannot be definitively established. Because deworming history was obtained through parent self‐report, the findings may be subject to recall bias.

## 5. Conclusions

This study provides valuable insights into the current prevalence and intensity of STH and SCH infections among preschool children in Tanzania, a population vulnerable to the detrimental effects of these parasites. The low overall prevalence of STH and SCH suggests a positive impact of the ongoing deworming program. However, the persistent prevalence of anemia, despite the low parasitic infection rates, indicates the potential influence of other contributing factors beyond helminth infections. The observed disparity in worm prevalence between rural and urban areas underscores the need for targeted interventions in rural communities. While parental knowledge of STH was relatively high, the reported deworming coverage suggests potential gaps in program implementation or access. These findings emphasize the importance of continuous monitoring and evaluation of deworming programs to ensure sustained effectiveness and identify areas for improvement. In parallel, integrated interventions including improved child nutrition, strengthened malaria control, and routine deworming are essential for reducing the combined burden of infection and anemia. Regular assessments of both parasitic infection and anemia prevalence are crucial for guiding public health strategies and optimizing child health outcomes in Tanzania.

## Author Contributions

Conceptualization and design of the study: K.A., A.K., D.M., F.M., T.E., R.M., M.K., P.K., D.T., and G.L. Data curation: K.A. and A.K. Formal analysis: K.A., A.K., and D.M. Investigation: K.A., A.K., D.M., F.M., and M.K. Methodology: K.A., A.K., D.M., R.M., and M.K. Project administration: A.K., F.M., T.E., D.T., and G.L. Resources: T.E., R.M., F.M., A.K., and G.L. Supervision: D.T. and G.L. Writing—original draft: K.A., A.K., and D.M. Writing—review and editing: F.M., T.E., R.M., M.K., P.K., D.T., and G.L. K.A. and A.K. have contributed to the work equally and should be regarded as cofirst authors.

## Funding

UNICEF Tanzania generously funded the operational expenses of the project, excluding publication processing costs.

## Disclosure

All authors read and approved the final manuscript.

## Ethics Statement

Ethical approval for conducting the survey was provided by the Tanzanian National Institute for Medical Research, Certificate No. NIMR/HQ/QR.8a/Vol. IX/4086. Written informed consent was obtained from all parents or legal guardians of preschool children after providing them with relevant study information. To ensure participant confidentiality, all data were anonymized using unique identification codes. No personal identifiers were included in the final analytic dataset. Electronic data were stored on password‐protected servers accessible only to authorized study personnel, and paper‐based records were kept in locked cabinets.

## Conflicts of Interest

The authors declare no conflicts of interest.

## Supporting information


**Supporting Information** Additional supporting information can be found online in the Supporting Information section. Table S1: Water, sanitation, and hygiene (WASH) practices. Table S2: Knowledge on STH and SCH. Table S3: History of vitamin A supplementation and STH and SCH treatment.

## Data Availability

The data that support the findings of this study are not publicly archived but are available from the corresponding author upon reasonable request.

## References

[1] Goshu A. , Alemu G. , and Ayehu A. , Prevalence and Intensity of Soil-Transmitted Helminths and Associated Factors Among Adolescents and Adults in Bibugn Woreda, Northwest Ethiopia: A Community-Based Cross-Sectional Study, Journal of Tropical Medicine. (2021) 2021, 7043881, 10.1155/2021/7043881, 34976073. 34976073 PMC8718294

[2] Pullan R. L. , Smith J. L. , Jasrasaria R. , and Brooker S. J. , Global Numbers of Infection and Disease Burden of Soil Transmitted Helminth Infections in 2010, Parasites & Vectors. (2014) 7, no. 1. 10.1186/1756-3305-7-37 PMC3905661 24447578

[3] Crompton D. W. T. and Nesheim M. C. , Nutritional Impact of Intestinal Helminthiasis During Thehumanlifecycle, Annual Review of Nutrition. (2002) 22, no. 1, 35–59, 10.1146/annurev.nutr.22.120501.134539, 2-s2.0-0036399683. 12055337

[4] Fauziah N. , Aviani J. K. , Agrianfanny Y. N. , and Fatimah S. N. , Intestinal Parasitic Infection and Nutritional Status in Children Under Five Years Old: A Systematic Review, Tropical Medicine and Infectious Disease. (2022) 7, no. 11, 10.3390/tropicalmed7110371. PMC9697828 36422922

[5] Hall A. , Hewitt G. , Tuffrey V. , and De S. N. , A Review and Meta-Analysis of the Impact of Intestinal Worms on Child Growth and Nutrition, Maternal & Child Nutrition. (2008) 4, 118–236, 10.1111/j.1740-8709.2007.00127.x, 2-s2.0-39449101115. 18289159 PMC6860651

[6] Masangcay D. U. , Amado A. J. Y. , Bulalas A. R. , Ciudadano S. R. , Fernandez J. D. , Sastrillo S. M. , and Mabaggu R. M. , Association of Soil-Transmitted Helminth Infection and Micronutrient Malnutrition: A Narrative Review, Asian Journal of Biological and Life Sciences. (2021) 10, no. 2, 317–324, 10.5530/ajbls.2021.10.44.

[7] Bartlett A. W. , Mendes E. P. , Dahmash L. , Palmeirim M. S. , de Almeida M. C. , Peliganga L. B. , Lufunda L. M. M. , Direito A. , Ramirez J. , Mwinzi P. N. , Lopes S. , and Vaz Nery S. , School-Based Preventive Chemotherapy Program for Schistosomiasis and Soil-Transmitted Helminth Control in Angola: 6-Year Impact Assessment, PLOS Neglected Tropical Diseases. (2023) 17, no. 5, e0010849, 10.1371/journal.pntd.0010849, 37196040. 37196040 PMC10228770

[8] Dyer C. E. F. , Ng-Nguyen D. , Clarke N. E. , Hii S. F. , Nguyen H. Q. , Nguyen V. A. T. , Nguyen T. V. , Nguyen T. V. , Ngo T. T. , Herath H. M. P. D. , Wand H. , Coffeng L. E. , Marshall J. C. , Gray D. J. , Anderson R. M. , Clements A. C. A. , Kaldor J. M. , Traub R. J. , and Vaz Nery S. , Community-Wide Versus School-Based Targeted Deworming for Soil-Transmitted Helminth Control in School-Aged Children in Vietnam: The CoDe-STH Cluster-Randomised Controlled Trial, Lancet Regional Health–Western Pacific. (2023) 41, 100920, 10.1016/j.lanwpc.2023.100920, 37860203. 37860203 PMC10583164

[9] Rite E. E. , Kapalata S. N. , and Munisi D. Z. , Prevalence, Intensity, and Factors Associated With Urogenital Schistosomiasis Among Women of Reproductive Age in Mbogwe District Council, Geita Region, Tanzania, BioMed Research International. (2020) 2020, no. 1, 5923025, 10.1155/2020/5923025, 33178830. 33178830 PMC7609139

[10] Franz A. , Fuss A. , Mazigo H. D. , Ruganuza D. , and Müller A. , Prevalence of Schistosoma mansoni, Soil-Transmitted Helminths and Intestinal Protozoa in Orphans and Street Children in Mwanza City, Northern Tanzania, Infection. (2023) 51, no. 5, 1399–1406, 10.1007/s15010-023-01999-9, 36805439. 36805439 PMC10545637

[11] Fuss A. , Mazigo H. D. , Tappe D. , Kasang C. , and Mueller A. , Comparison of Sensitivity and Specificity of Three Diagnostic Tests to Detect Schistosoma mansoni Infections in School Children in Mwanza region, Tanzania, PLoS One. (2018) 13, no. 8, 1–14, 10.1371/journal.pone.0202499, 2-s2.0-85052096645. PMC6105001 30133490

[12] Molvik M. , Helland E. , Zulu S. G. , Kleppa E. , Lillebo K. , Gundersen S. G. , Kvalsvig J. D. , Taylor M. , Kjetland E. F. , and Vennervald B. J. , Co-Infection With Schistosoma haematobium and Soil-Transmitted Helminths in Rural South Africa, South African Journal of Science. (2017) 113, no. 3-4, 1–6, 10.17159/sajs.2017/20160251, 2-s2.0-85017558510.

[13] World Health Organization , Fifty-Fourth World Health Assembly: Resolutions and Decisions, 2001, WHO.

[14] Moshi C. C. , Sebastian P. J. , Azizi K. A. , Killel E. , Mushumbusi D. G. , Meghji W. P. , Kitunda M. E. , Millinga F. K. , Adam H. , and Kasankala L. M. , Effect of Deworming on Health Outcomes among Children Aged 12–59 Months in Tanzania: A Multilevel Mixed Effects Analysis, Journal of Nutrition and Metabolism. (2023) 2023, 9529600, 10.1155/2023/9529600. 37520400 PMC10382239

[15] Said K. , Hella J. , Knopp S. , Nassoro T. , Shija N. , Aziz F. , Mhimbira F. , Schindler C. , Mwingira U. , Mandalakas A. M. , Manji K. , Tanner M. , Utzinger J. , and Fenner L. , Schistosoma, Other Helminth Infections, and Associated Risk Factors in Preschool-Aged Children in Urban Tanzania, PLoS Neglected Tropical Diseases. (2017) 11, no. 11, e0006017, 10.1371/journal.pntd.0006017, 2-s2.0-85036657800, 29108003. 29108003 PMC5697890

[16] World Health Organization , Prevention and Control of Schistosomiasis and Soil-Transmitted Helminthiasis, World Health Organization Technical Report Series. (2002) 912. 12592987

[17] World Health Organization , Haemoglobin Concentrations for the Diagnosis of Anaemia and Assessment of Severity, 2011, World Health Organization, http://scholar.google.com/scholar?hl=en%26btnG=Search%26q=intitle:Haemoglobin+concentrations+for+the+diagnosis+of+anaemia+and+assessment+of+severity#1.

[18] Sullivan K. M. , Mei Z. , Grummer-Strawn L. , and Parvanta I. , Haemoglobin Adjustments to Define Anaemia, Tropical Medicine & International Health. (2008) 13, no. 10, 1267–1271, 10.1111/j.1365-3156.2008.02143.x, 2-s2.0-53549127247. 18721184

[19] Eltantawy M. , Orsel K. , Schroeder A. , Morona D. , Mazigo H. D. , Kutz S. , Hatfield J. , Manyama M. , and van der Meer F. , Soil Transmitted Helminth Infection in Primary School Children Varies With Ecozone in the Ngorongoro Conservation Area, Tanzania, Tropical Medicine and Health. (2021) 49, no. 1, 10.1186/s41182-021-00310-6. PMC7945338 33691800

[20] Siza J. E. , Kaatano G. M. , Chai J.-Y. , Eom K. S. , Rim H.-J. , Yong T.-S. , Min D. Y. , Chang S. Y. , Ko Y. , and Changalucha J. M. , Prevalence of Schistosomes and Soil-Transmitted Helminths Among Schoolchildren in Lake Victoria Basin, Tanzania, Korean Journal of Parasitology. (2015) 53, no. 5, 515–524, 10.3347/kjp.2015.53.5.515, 2-s2.0-84945971141, 26537030. 26537030 PMC4635830

[21] Ame S. , Kabole F. , Nanai A. M. , Mwinzi P. , Mupfasoni D. , Ali S. M. , and Montresor A. , Impact of Preventive Chemotherapy on Transmission of Soil-Transmitted Helminth Infections in Pemba Island, United Republic of Tanzania, 1994–2021, PLoS Neglected Tropical Diseases. (2022) 16, no. 6, e0010477, 10.1371/journal.pntd.0010477, 35759453. 35759453 PMC9236265

[22] Wammes L. J. , Hamid F. , Wiria A. E. , May L. , Kaisar M. M. M. , Prasetyani-Gieseler M. A. , Djuardi Y. , Wibowo H. , Kruize Y. C. M. , Verweij J. J. , de Jong S. E. , Tsonaka R. , Houwing-Duistermaat J. J. , Sartono E. , Luty A. J. F. , Supali T. , and Yazdanbakhsh M. , Community Deworming Alleviates Geohelminth-Induced Immune Hyporesponsiveness, Proceedings of the National Academy of Sciences. (2016) 113, no. 44, 12526–12531, 10.1073/pnas.1604570113, 2-s2.0-84993990774, 27791067. PMC5098677 27791067

[23] Brooker S. , Alexander N. , Geiger S. , Moyeed R. A. , Stander J. , Fleming F. , Hotez P. J. , Correa-Oliveira R. , and Bethony J. , Contrasting Patterns in the Small-Scale Heterogeneity of Human Helminth Infections in Urban and Rural Environments in Brazil, International Journal for Parasitology. (2006) 36, no. 10–11, 1143–1151, 10.1016/j.ijpara.2006.05.009, 2-s2.0-33747118325, 16814294. 16814294 PMC1783908

[24] World Health Organization , Ending the Neglect to Attain the Sustainable Development Goals: A Road Map for Neglected Tropical Diseases 2021–2030, 2020, World Health Organization.

[25] Rajagopal S. , Hotez P. J. , and Bundy D. A. P. , Micronutrient Supplementation and Deworming in Children With Geohelminth Infections, PLOS Neglected Tropical Diseases. (2014) 8, no. 8, e2920, 10.1371/journal.pntd.0002920, 2-s2.0-84929354064. 25101963 PMC4125305

[26] Clohossey P. C. , Katcher H. I. , Mogonchi G. O. , Nyagoha N. , Isidro M. C. , Kikechi E. , Okoth E. E. , and Blankenship J. L. , Coverage of Vitamin A Supplementation and Deworming During Malezi Bora in Kenya, Journal of Epidemiology and Global Health. (2014) 4, no. 3, 169–176, 10.1016/j.jegh.2013.12.005, 2-s2.0-84905594230, 25107652. 25107652 PMC7333821

[27] Laillou A. , Baye K. , Zelalem M. , and Chitekwe S. , Vitamin A Supplementation and Estimated Number of Averted Child Deaths in Ethiopia: 15 Years in Practice (2005–2019), Maternal & Child Nutrition. (2021) 17, no. 3, e13132, 10.1111/mcn.13132. 33336556 PMC8189216

[28] Anah M. U. , Ikpeme O. E. , Etuk I. S. , Yong K. E. , Ibanga I. , and Asuquo B. E. , Worm Infestation and Anaemia Among Pre-School Children of Peasant Farmers in Calabar, Nigeria, Nigerian Journal of Clinical Practice. (2008) 11, no. 3, 220–224, 19140358. 19140358

[29] Premji Z. , Hamisi Y. , Shiff C. , Minjas J. , Lubega P. , and Makwaya C. , Anaemia and Plasmodium falciparum Infections Among Young Children in an Holoendemic Area, Bagamoyo, Tanzania, Acta Tropica. (1995) 59, no. 1, 55–64, 10.1016/0001-706X(94)00079-G, 2-s2.0-0028968818, 7785526. 7785526

[30] Mchau G. , Masanja H. , Killel E. , Azizi K. , Lukindo T. , Hancy A. , Henry S. , Paul H. , Sanga A. , Mwiru R. , and Zvandaziva C. , Micronutrient Deficiencies and Their Cooccurrence Among Pregnant Women in Mbeya Region, Tanzania, PLoS One. (2024) 19, no. 11, e0309620, 10.1371/journal.pone.0309620, 39556557. 39556557 PMC11573179

[31] John S. E. , Azizi K. , Hancy A. , Twin’omujuni A. , Katana D. , Shine J. , Lyatuu V. , Sanga A. , Mwiru R. S. , Abdallah F. , and Mchau G. , The Prevalence and Risk Factors Associated With Iron, Vitamin B12 and Folate Deficiencies in Pregnant Women: A Cross-Sectional Study in Mbeya, Tanzania, PLOS Global Public Health. (2023) 3, no. 4, e0001828, 10.1371/journal.pgph.0001828, 37083656. 37083656 PMC10121015

[32] Kiani A. K. , Dhuli K. , Donato K. , Aquilanti B. , Velluti V. , and Matera G. , Main Nutritional Deficiencies, Journal of Preventive Medicine and Hygiene. (2022) 63, no. 2, E93–101, 10.15167/2421-4248/jpmh2022.63.2S3.2752. 36479498 PMC9710417

[33] Ambrose E. E. , Makani J. , Chami N. , Masoza T. , Kabyemera R. , Peck R. N. , Kamugisha E. , Manjurano A. , Kayange N. , and Smart L. R. , High Birth Prevalence of Sickle Cell Disease in Northwestern Tanzania, Pediatric Blood & Cancer. (2018) 65, no. 1, e26735, 10.1002/pbc.26735, 2-s2.0-85026644134. PMC5701733 28766840

[34] Ambrose E. E. , Smart L. R. , Charles M. , Hernandez A. G. , Latham T. , Hokororo A. , Beyanga M. , Howard T. A. , Kamugisha E. , McElhinney K. E. , Tebuka E. , and Ware R. E. , Surveillance for Sickle Cell Disease, United Republic of Tanzania, Bulletin of the World Health Organization. (2020) 98, no. 12, 859–868, 10.2471/BLT.20.253583. 33293746 PMC7716099

[35] Gangat N. and Wolanskyj A. P. , Anemia of Chronic Disease, Seminars in Hematology. (2013) 50, no. 3, 232–238, 10.1053/j.seminhematol.2013.06.006, 2-s2.0-84881600486. 23953340

[36] Twum S. , Fosu K. , Felder R. A. , and Sarpong K. A. N. , Bridging the Gaps in Newborn Screening Programmes: Challenges and Opportunities to Detect Haemoglobinopathies in Africa, African Journal of Laboratory Medicine. (2023) 12, no. 1, 10.4102/ajlm.v12i1.2225. PMC10729498 38116518

[37] Kent S. , Weinberg E. D. , and Stuart-Macadam P. , The Etiology of the Anemia of Chronic Disease and Infection, Journal of Clinical Epidemiology. (1994) 47, no. 1, 23–33, 10.1016/0895-4356(94)90030-2, 2-s2.0-0028055056. 8283191

[38] Gebreegziabher T. and Sidibe S. , Prevalence and Contributing Factors of Anaemia Among Children Aged 6-24 Months and 25-59 Months in Mali, Journal of Nutritional Science. (2023) 12, 10.1017/jns.2023.93. PMC10641697 37964977

