# Supplementary material for: Prevalence and Infection Intensity of Soil‐Transmitted Helminths and Schistosomiasis Among Preschool Children in Mainland Tanzania
Source: J Parasitol Res. 2026 Apr 22;2026:6492754. doi: 10.1155/japr/6492754 (PMC13100894; doi:10.1155/japr/6492754)
Supplement: Supplementary file 1 — Supporting Information Additional supporting information can be found online in the Supporting Information section. Table S1: Water, sanitation, and hygiene (WASH) practices. Table S2: Knowledge on STH and SCH. Table S3: History of vitamin A supplementation and STH and SCH treatment. [file JAPR-2026-6492754-s001.docx]

**SUPPLEMENTARY TABLES**

S1 Table. Water, sanitation and hygiene (WASH) practices

| **Variable** | **Frequency (n)** | **Percentage (%)** |
| --- | --- | --- |
| **Source of drinking water (N = 806)** |  |  |
| **Improved water sources** | **704** | **87.3** |
| Piped to dwelling/yard/plot | 192 | 23.8 |
| Piped to neighbor | 147 | 18.2 |
| Public tap/standpipe | 193 | 23.9 |
| Tubewell/borehole | 27 | 3.3 |
| Protected dugwell | 123 | 15.3 |
| Protected Spring | 18 | 2.2 |
| Rainwater | 2 | 0.2 |
| Bottled water | 2 | 0.2 |
| **Unimproved water sources** | **102** | **12.7** |
| Unprotected dugwell | 53 | 6.6 |
| Unprotected spring | 7 | 0.9 |
| Tanker truck/cart with small tank | 22 | 2.7 |
| Surface water | 20 | 2.5 |
| Time to go and fetch water |  |  |
| Minutes | 771 | 95.7 |
| Hours | 35 | 4.3 |
| **Sanitation facilities (N = 806)** |  |  |
| **Type of toilet used in HH** |  |  |
| **Improved sanitation facility** | 376 | 46.7 |
| Flush/pour flush to pipe sewer system | 10 | 1.2 |
| Flush/pour flush to septic tank | 61 | 7.6 |
| Flush/pour flush to pit latrine | 89 | 11 |
| Ventilated improved pit latrine | 94 | 11.7 |
| Pit latrine with slab | 222 | 27.5 |
| Compositing toilet | 1 | 0.1 |
| **Unimproved sanitation facility** | 430 | 53.3 |
| Flush/pour to flush to other areas | 14 | 1.7 |
| Pit latrine without slab/open pit bucket | 307 | 38.1 |
| Open defecation/no facility/bush/field | 8 | 1 |
| Sharing toilets with other HH |  |  |
| Yes | 134 | 16.6 |
| No | 672 | 83.4 |
| **Hygiene Practices (N=1,612)** |  |  |
| Where the child defecates |  |  |
| Toilet | 807 | 50.0 |
| Potty | 332 | 20.6 |
| Assisted potting(Ground/floor) | 175 | 10.9 |
| In hole | 10 | 0.6 |
| Diaper/clothes | 45 | 2.8 |
| On the ground(open space) | 243 | 15.1 |
| Children wearing shoes when visiting the toilet?** |  |  |
| All the time | 470 | 39.3 |
| Sometimes | 467 | 39.0 |
| No, seldom | 108 | 9.0 |
| Never | 151 | 12.6 |
| **Hand wash practice** |  |  |
| Handwashing after using toilets |  |  |
| NO | 861 | 53.4 |
| YES | 751 | 46.6 |
| Handwashing after playing |  |  |
| NO | 1440 | 89.3 |
| YES | 172 | 10.7 |
| Handwashing before eating |  |  |
| NO | 133 | 8.3 |
| YES | 1479 | 91.7 |
| Handwashing after eating |  |  |
| NO | 254 | 15.8 |
| YES | 1358 | 84.2 |
| What is used for handwashing? |  |  |
| Water alone | 641 | 37.8 |
| Water and detergent | 951 | 59.0 |
| Other | 20 | 1.2 |

*Improved water sources: Piped water into dwelling, piped water to yard/plot, public water or stand pipe, tube well or borehole, protected dug well, protected spring, bottled water, rainwater

*Unimproved water sources: unprotected dug well, unprotected spring, cart with small tank/drum, tanker truck, surface water

** totals differ due to missing values in some variables

S2 Table: Knowledge on STH and SCH (N=806)

| **Variable** | **Frequency (n)** | **Percentage (%)** |
| --- | --- | --- |
| **Knowledge on STH** |  |  |
| Yes | 687 | 85.2 |
| No | 119 | 14.8 |
| **Source of information on STH*** |  |  |
| Radio | 15 | 2.2 |
| Billboard | 1 | 0.1 |
| Magazines | 1 | 0.1 |
| Healthcare worker | 554 | 80.6 |
| Community health worker | 84 | 12.2 |
| Media | 1 | 0.1 |
| Other | 31 | 4.5 |
| **Knowledge on schistosomiasis** |  |  |
| Yes | 462 | 57.3 |
| No | 344 | 42.7 |
| **Source of information on Schistosomiasis*** |  |  |
| Radio | 27 | 5.8 |
| Television | 2 | 0.4 |
| Billboard | 1 | 0.2 |
| Magazines | 1 | 0.2 |
| Healthcare worker | 299 | 64.7 |
| Community health worker | 49 | 10.6 |
| Media | 12 | 2.6 |
| Other | 71 | 15.4 |

*totals differ due to missing values in some variables

S3 Table: History of vitamin A supplementation, STH and SCH treatment

| **Variable** | **Frequency (n)** | **Percentage (%)** |
| --- | --- | --- |
| Vitamin A supplementation in last 6 month |  |  |
| Yes | 1316 | 81.64 |
| No | 266 | 16.50 |
| I don’t know | 30 | 1.86 |
| Ever given deworming drug to your child |  |  |
| Yes | 928 | 57.57 |
| No | 449 | 27.85 |
| Don't remember | 235 | 14.58 |
| Last time a child received deworming tablet (months) |  |  |
| 0 - 3 | 510 | 54.96 |
| 4 - 6 | 275 | 29.63 |
| >6 | 143 | 15.41 |
| Ever given anti-schistosomal drugs* |  |  |
| Yes | 8 | 0.88 |
| No | 904 | 99.12 |
| Last time a child anti-schistosomal drugs (months) |  |  |
| 0-3 | 3 | 37.50 |
| 4-6 | 1 | 12.50 |
| ˃6 | 4 | 50.00 |

*only those who were aware of schistosomiasis

rem
